# Supplementary material for: Killiverse: an interactive multi-omics web resource for killifish
Source: bioRxiv. 2026 Jun 21:2026.06.16.731504. Preprint. [Version 1] doi: 10.64898/2026.06.16.731504 (PMC13307987; doi:10.64898/2026.06.16.731504)

Supplementary Data

Table S1: Studies with links to Killiverse, GEO accession numbers for the datasets, assay type, scientific name of the species and strain

No.	Study title with hyperlink to Killiverse	GEO accession of dataset	RNA-seq modality	Species (strain)
1.	Multi-tissue transcriptomic aging atlas reveals predictive aging biomarkers in the killifish	PRJNA1274512	bulk RNA-seq (6 age groups)	*N. furzeri* (GRZ)
2.	Vertebrate diapause preserves organisms long term through Polycomb complex members	PRJNA503701	bulk RNA-seq	*N. furzeri* (GRZ)
3.	Widespread sex dimorphism across single-cell transcriptomes of adult African turquoise killifish tissues[a]	PRJNA952805	single cell RNA-seq	*N. furzeri* (GRZ)
4.	Transcriptomes of aging brain, heart, muscle, and spleen from female and male African turquoise killifish	SRP430823 (PRJNA952180)	bulk RNA-seq	*N. furzeri* (GRZ)
5.	The killifish germline regulates longevity and somatic repair in a sex-specific manner	PRJNA1045681	bulk RNA-seq	*N. furzeri* (GRZ)
6.	Regulation of life span by the gut microbiota in the short-lived African turquoise killifish	PRJNA379208	bulk RNA-seq	*N. furzeri* (GRZ)
7.	An automated feeding system for the African killifish reveals effects of dietary restriction on lifespan and allows scalable assessment of associative learning	GSE216369 (PRJNA893228)	bulk RNA-seq	*N. furzeri* (GRZ)
8.	Refeeding-associated AMPKγ1 complex activity is a hallmark of health and longevity	PRJNA817434	bulk RNA-seq	*N. furzeri* (GRZ)
9.	Genetic perturbation of AMP biosynthesis extends lifespan and restores metabolic health in a naturally short-lived vertebrate	GSE190757 (PRJNA788399)	bulk RNA-seq	*N. furzeri* (GRZ)
10.	RNA-seq of the aging brain in the short-lived fish N. furzeri - conserved pathways and novel genes associated with neurogenesis	GSE52462 (PRJNA229052)	bulk RNA-seq (5 age groups)	*N. furzeri* (MZM-04/10)

11.	Evolution of diapause in the African turquoise killifish by remodeling the ancient gene regulatory landscape (Dataset 1)	GSE185817 (PRJNA770943)	bulk RNA-seq	*N. furzeri* (GRZ)
12.	Evolution of diapause in the African turquoise killifish by remodeling the ancient gene regulatory landscape (Dataset 2)	Supplemental information S7	lipidomics	*N. furzeri* (GRZ)
13.	Evolution of diapause in the African turquoise killifish by remodeling the ancient gene regulatory landscape (Dataset 3)	Supplemental information S7	lipidomics	*N. furzeri* (GRZ) *A. striatum* (Aquarium)
14.	MicroRNA miR-29 controls a compensatory response to limit neuronal iron accumulation during adult life and aging	GSE79825 (PRJNA317074)	bulk RNA-seq	*N. furzeri* (MZM-04/10)
15.	Reduced proteasome activity in the aging brain results in ribosome stoichiometry loss and aggregation	GSE150318 (PRJNA631760)	bulk RNA-seq	*N. furzeri* (MZM-04/10)
16.	Aging triggers H3K27 trimethylation hoarding in the chromatin of Nothobranchius furzeri skeletal muscle	GSE135032 (PRJNA557199)	bulk RNA-seq	*N. furzeri* (MZM-04/10)
17.	Dynamic regulation of gonadal transposon control across the lifespan of the naturally short-lived African turquoise killifish	PRJNA854614	bulk RNA-seq	*N. furzeri* (GRZ)
18.	Circular RNAs in the ageing African turquoise killifish	GSE134065 (PRJNA553674)	bulk RNA-seq	*N. furzeri* (GRZ)
19.	The effect of meclofenoxate on the transcriptome of aging brain of Nothobranchius guentheri annual killifish	PRJNA779252	bulk RNA-seq	*N. guentheri* (Zanzibar Tan 14-02)
20.	Exercise datasets[b]	PRJNA407099 (muscle), PRJNA407101 (skin), PRJNA407084 (brain), PRJNA407091 (liver)	bulk RNA-seq	*N. furzeri* (MZM-04/10)

21.	Longitudinal RNA-seq analysis of vertebrate aging identifies mitochondrial complex I as a small-molecule-sensitive modifier of lifespan (Dataset I)	GSE66712 (PRJNA277768)	bulk RNA-seq	*N. furzeri* (MZM-04/10)
22.	Longitudinal RNA-seq analysis of vertebrate aging Identifies mitochondrial complex I as a small-molecule-sensitive modifier of lifespan (Dataset II)	GSE66712 (PRJNA277768)	bulk RNA-seq (5 age groups)	*N. furzeri* (MZM-04/10)
23.	RNAseq analysis of brain aging in wild specimens of short-lived turquoise killifish: Commonalities and differences with aging under laboratory conditions (Dataset I)	GSE183039 (PRJNA758914)	bulk RNA-seq	*N. furzeri* (MZM-04/10)
24.	RNAseq Analysis of Brain Aging in Wild Specimens of short-lived turquoise killifish: Commonalities and differences with aging under laboratory conditions (Dataset II)	GSE183039 (PRJNA758914)	bulk RNA-seq	*N. furzeri* (MZM-04/10), *N. furzeri* (MZM-A41),
25.	Age-related dysregulation of the retinal transcriptome in African turquoise killifish	GSE255363 (PRJNA1074746)	bulk RNA-seq	*N. furzeri* (GRZ)
26.	Changes in regeneration-responsive enhancers shape regenerative capacities in vertebrates	PRJNA559885	bulk RNA-seq	*N. furzeri* (GRZ)
27.	Analysis of different strains of the turquoise killifish *Nothobranchius furzeri* identifies transcriptomic signatures associated with heritable lifespan differences	GSE103132 (PRJNA400236)	bulk RNA-seq (4 age groups)	*N. furzeri* (GRZ)
28.	Temperature-dependent vitamin D signaling regulates developmental trajectory associated with diapause in an annual killifish [c]	PRJNA272154	bulk RNA-seq	*A. limnaeus* (Quisiro)
29.	Single housing of juveniles accelerates early-stage growth but extends adult lifespan in African turquoise killifish (Dataset I)	GSE245483 (PRJNA1028576)	bulk RNA-seq (5 age groups)	*N. furzeri* (GRZ)
30.	Single housing of juveniles accelerates early-stage growth but extends adult lifespan in African turquoise killifish (Dataset II)	GSE245483 (PRJNA1028576)	bulk RNA-seq (5 age groups)	*N. furzeri* (GRZ)

31.	Single housing of juveniles accelerates early-stage growth but extends adult lifespan in African turquoise killifish (Dataset III)	GSE245483 (PRJNA1028576)	bulk RNA-seq	*N. furzeri* (GRZ)
32.	The killifish germline regulates longevity and somatic repair in a sex-specific manner (Dataset I)	GSE248741 (PRJNA1045681)	single cell RNA-seq	*N. furzeri* (GRZ)
33.	The killifish germline regulates longevity and somatic repair in a sex-specific manner (Dataset II)	GSE248741 (PRJNA1045681)	single cell RNA-seq	*N. furzeri* (GRZ)
34.	The killifish germline regulates longevity and somatic repair in a sex-specific manner (Dataset III)	GSE248741 (PRJNA1045681)	single cell RNA-seq	*N. furzeri* (GRZ)
35.	The killifish germline regulates longevity and somatic repair in a sex-specific manner (Dataset IV)	GSE248741 (PRJNA1045681)	single cell RNA-seq	*N. furzeri* (GRZ)
36.	The genome of *Austrofundulus limnaeus* offers insights into extreme vertebrate stress tolerance and embryonic development	PRJNA272154	bulk RNA-seq	*A. limnaeus* (Quisiro)
37.	Spontaneous onset of cellular markers of inflammation and genome instability during aging in the immune niche of the naturally short-lived turquoise killifish (*Nothobranchius furzeri*)	BioStudies S-BSST2265	single cell RNA-seq	*N. furzeri* (GRZ)
38.	Sodium-glucose co-transporter 2 inhibition improves age-dependent kidney microvascular rarefaction	PRJNA1265492	single nucleus RNA-seq	*N. furzeri* (GRZ)
39.	Tissue-specific landscape of protein aggregation and quality control in an aging vertebrate (Dataset 1)	Supplemental information Table S2	proteomics	*N. furzeri* (GRZ)
40.	Tissue-specific landscape of protein aggregation and quality control in an aging vertebrate (Dataset 2)	Supplemental information Table S2	proteomics	*N. furzeri* (GRZ)
41.	Tissue-specific landscape of protein aggregation and quality control in an aging vertebrate (Dataset 3)	Supplemental information Table S2	proteomics	*N. furzeri* (GRZ)

42.	A multi-omic atlas in the African turquoise killifish reveals increased glucocorticoid signaling as a hallmark of brain aging [d]	PRJNA1105049	single nucleus RNA-seq	*N. furzeri* (GRZ), *N. furzeri* (ZMZ-1001)
43.	Insights into Sex Chromosome Evolution and Aging from the Genome of a Short-Lived Fish	PRJEB5837	bulk RNA-seq	*N. furzeri* (GRZ), *N. furzeri* (MZM-0403)
44.	The human and non-human primate developmental GTEx projects [e]	dbGaP accession phs000424 (v10.p2)	bulk RNA-seq (6 age groups)	*Homo sapiens*
45.	Ageing hallmarks exhibit organ-specific temporal signatures	GSE132040 (PRJNA544748)	bulk RNA-seq (10 age groups)	*Mus musculus*

a. Seurat object available here: https://figshare.cosm/articles/dataset/Annotated_Seurat_object_containing_cells_for_a_single-cell_transcriptomic_atlas_of_adult_female_and_male_African_turquoise_killifish_/22766894
b. Study not published
c. Processed using GCF_001266775.1 *A. limnaeus* genome assembly.
d. Seurat object available here: https://figshare.com/articles/dataset/Annotated_Seurat_object_for_a_single-nuclei_transcriptomic_atlas_of_aging_female_and_male_African_turquoise_killifish_brains_of_the_GRZ_and_ZMZ-1001_strains/31847155?file=63065740
e. Raw count files and TPM files were obtained from gtexportal.org (https://gtexportal.org/home/downloads/adult-gtex/bulk_tissue_expression).

Ortholog mapping using reciprocal best BLAST hits

Table S2. List of species and the genome assemblies used for ortholog identification.

**Common name**	**Scientific name**	**Genome assembly**
Human	*Homo sapiens*	GCF_000001405.40
Mouse	*Mus musculus*	GCF_000001635.27
Zebrafish	*Danio rerio*	GCF_049306965.1
Japanese rice fish	*Oryzias latipes*	GCF_002234675.1
African turquoise killifish	*Nothobranchius furzeri*	GCF_001465895.1
Chicken	*Gallus gallus*	GCF_016699485.2
Rat	*Rattus norvegicus*	GCF_036323735.1
South American killifish	*Austrofundulus limnaeus*	GCF_001266775.1

Protein FASTA files were obtained from NCBI for the reference genome assemblies listed above. FASTA files were filtered to retain the longest protein sequence for each gene. BLASTP (version 2.16.0+) was run twice for every species. First, *N. furzeri's* protein FASTA file was used as the subject and the other species' protein FASTA file as the query. In the second run, *N. furzeri's* protein FASTA file was used as the query and the other species' protein FASTA file as the subject. E-value threshold was set to 0.01. BLASTP output files containing results for protein-protein sequence similarity searches were filtered to retain only the top hits (lowest e-value) for each gene. If both *N. furzeri* and the other species shared a pair of genes as the top hit, then they were considered orthologs. If no bidirectional hit was found, the top BLASTP hit obtained with *N. furzeri* as the query against other species was treated as the ortholog. Orthologs were identified for 22,119 out of 22,207 protein coding genes of *N. furzeri* in at least one of the other species.

Table S3. Full names of all the cell types in killifish and brain from Figure 4.

Mouse cells		Killifish cells	
**Abbreviation**	**Full name**	**Abbreviation**	**Full name**
ABC	Arachnoid barrier cells	AST	Astrocytes radial glia
ARP	Astrocyte-restricted precursors	EC	Ependymal cells
ASC	Astrocytes	ERY	Erythrocytes
CPC	Choroid plexus epithelial cells	GABA-N	GABAergic neurons
DC	Dendritic cells	GE Neuron	Granule excitatory neurons
EC	Endothelial cells	Microglia	Microglia
EPC	Ependymocytes	NSPC	NSPCs
Hb-VC	Hemoglobin-expressing vascular cells	Neuron-1	Neurons misc 1
HypEPC	Hypendymal cells	Neuron-2	Neurons misc 2
MAC	Macrophages	Neuron-3	Neurons misc 3
Microglia	Microglia	Neuron-4	Neurons misc 4
MNC	Monocytes	OPC	Oligodendrocyte progenitor cells
NendC	Neuroendocrine cells	OLG	Oligodendrocytes
ImmN	Immature neurons	PVI	PV interneurons
mNEUR	Mature neurons	PC	Purkinje cells
NEUT	Neutrophils	VSMC	Vascular smooth muscle cells
NRP	Neuronal restricted precursors		
NSC	Neural stem cells		
OEG	olfactory ensheathing glia		
OLG	oligodendrocytes		
OPC	oligodendrocyte precursor cells		
PC	Pericytes		
TNC	Tanycytes		
VLMC	Vascular and leptomeningeal cells		
VSMC	Vascular smooth muscle cells		

Figure S1. Killifish datasets filtered by library preparation protocol “Bulk RNA-seq” and condition “diapause”.

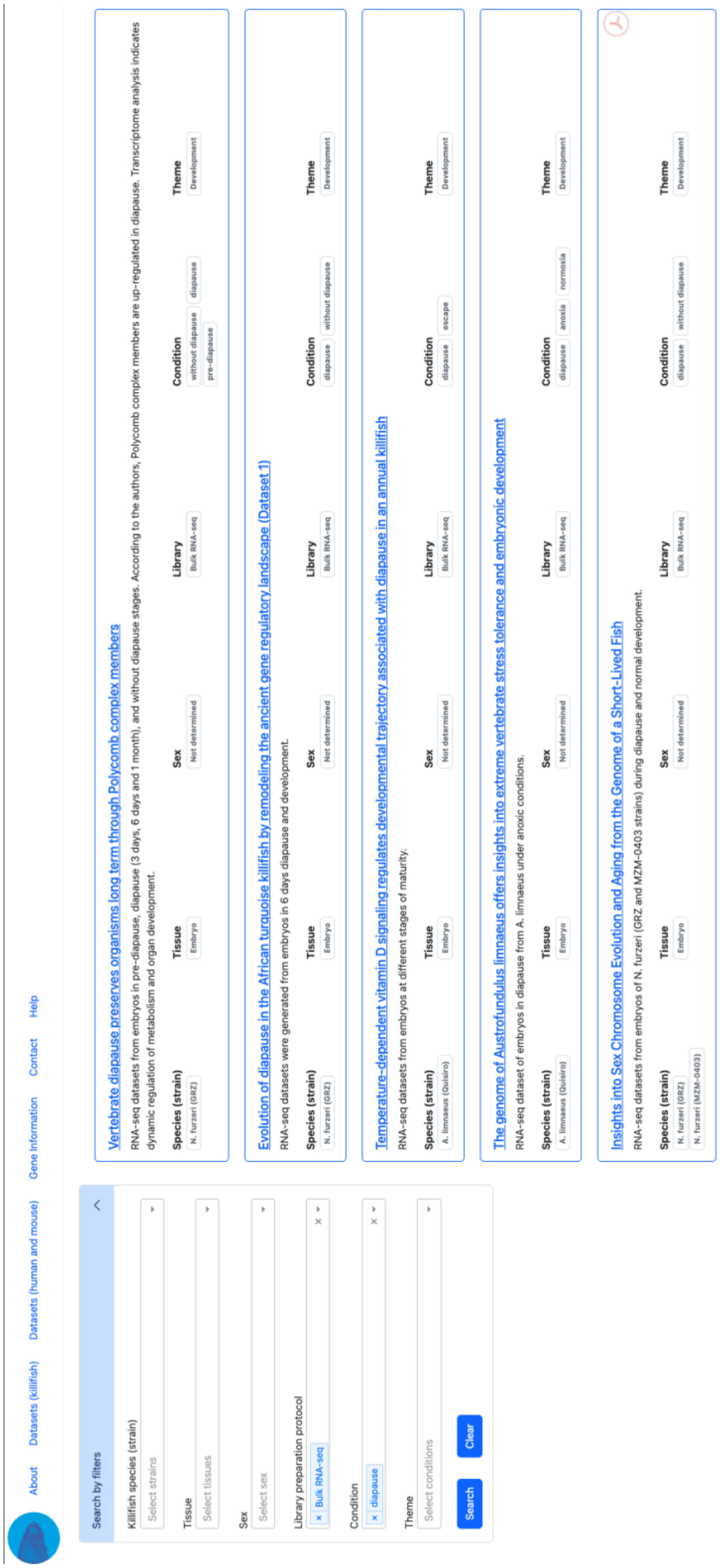

Figure S2. Box plot for the expression of *ezh1* and *ezh2* during pre-diapause (PreD), diapause 3-day (D3d), diapause 6-day (D6d), diapause 1-month (D1m) and non-diapause (NonD). Expression of *ezh1* is consistently higher in samples from diapause stage as compared to those in pre-diapause and non-diapause stages. The opposite is true for *ezh2*. This is part of the study [Vertebrate diapause preserves organisms long term through Polycomb complex members](#).

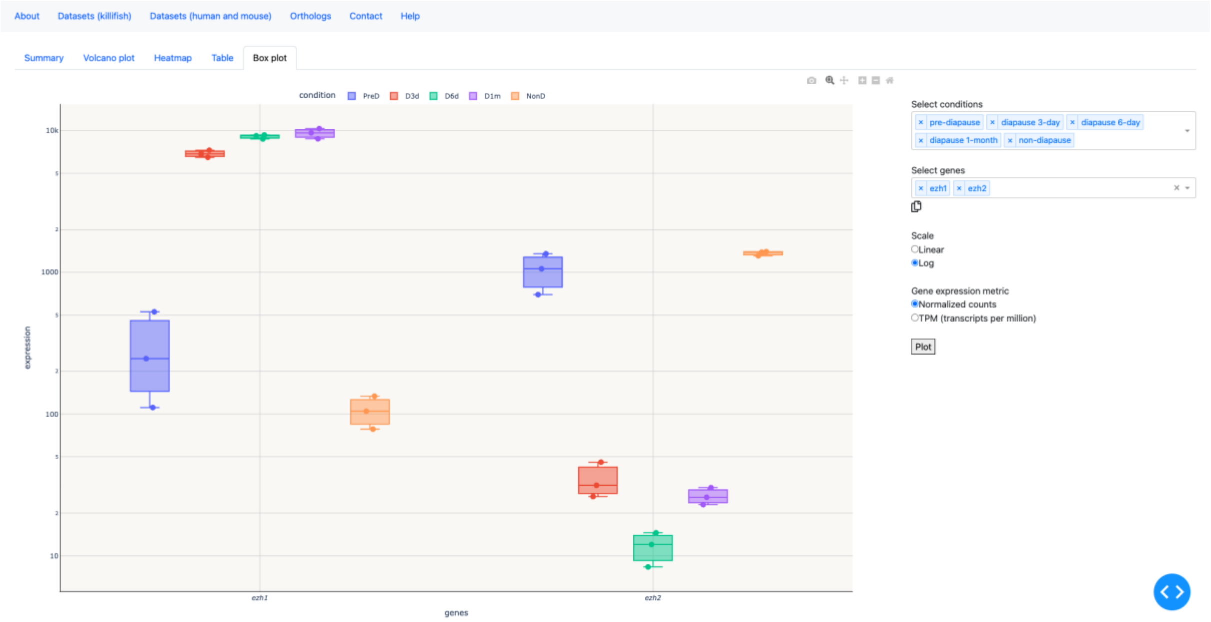

Figure S3. Heatmap for the expression of *ezh1* and *ezh2* genes during diapause (6 days) and development. Expression of *ezh1* tends to be higher in samples from diapause stage as compared to those development stage. The opposite is true for *ezh2*. This is part of the study [Evolution of diapause in the African turquoise killifish by remodeling the ancient gene regulatory landscape].

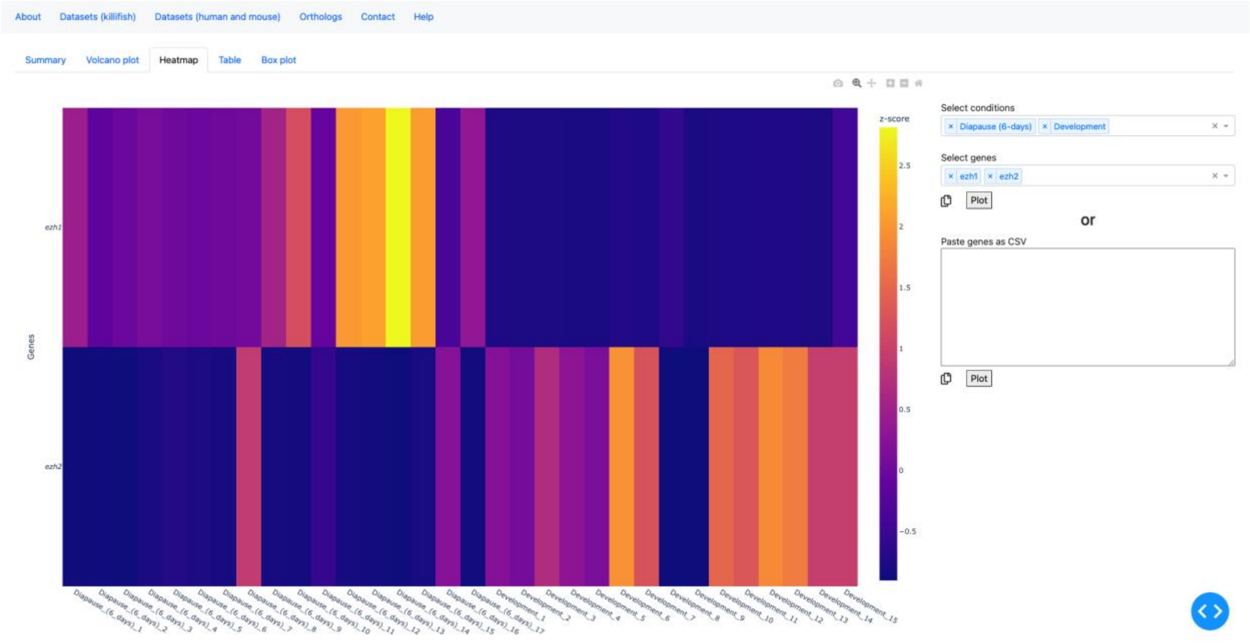

Figure S4. In the study titled Temperature-dependent vitamin D signaling regulates developmental trajectory associated with diapause in an annual killifish, embryos incubated at 20 °C committed to diapause trajectory while those incubated at 30 °C underwent normal development (escape trajectory). Expression of *ezh1* increased while that of *ezh2* declined as the embryo developed from dispersed cell stage (stage 1) to 24-pair somite stage (stage 7) during diapause. The opposite was true during normal development.

A. Expression of *ezh1* during diapause and normal development (escape).

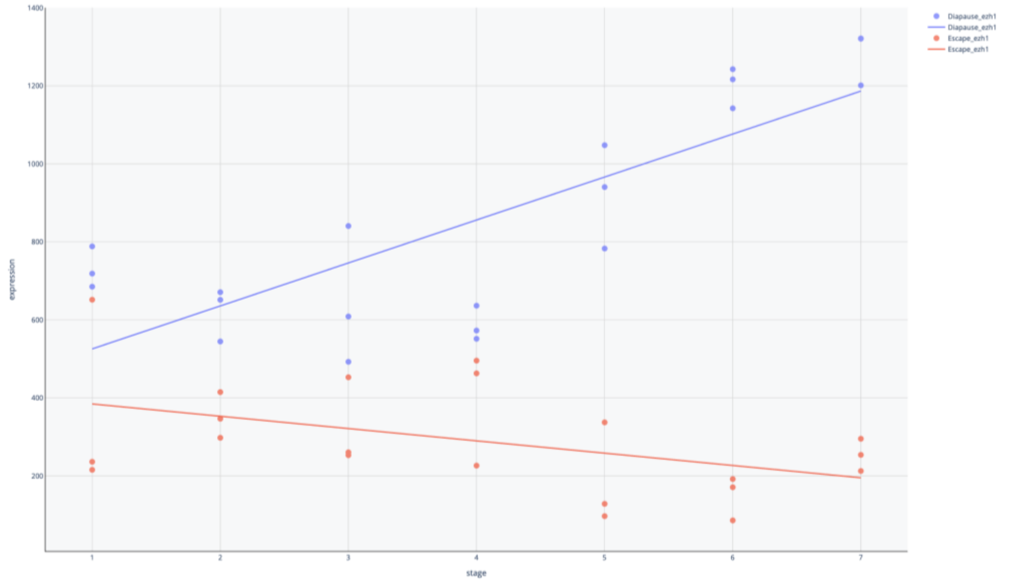

B. Expression of *ezh2* during diapause and normal development (escape).

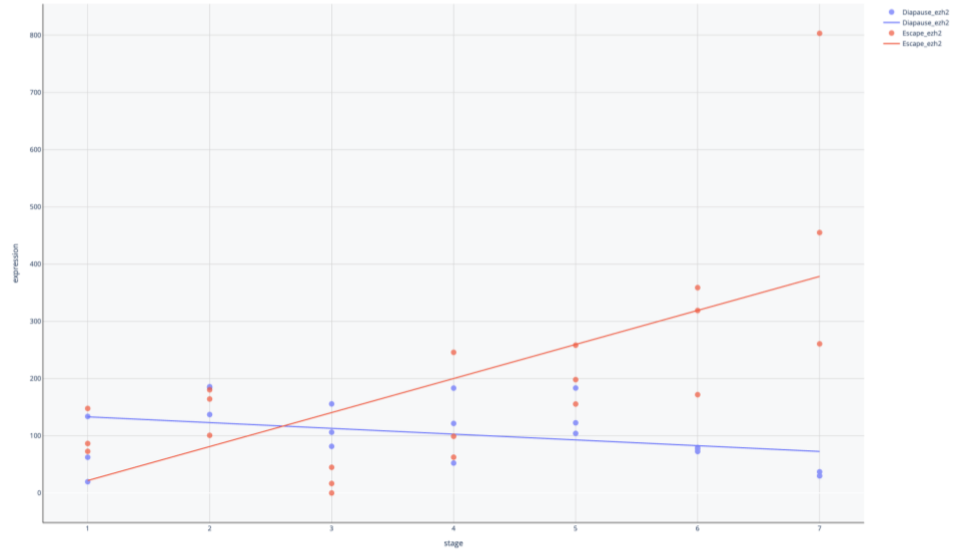

Figure S5. In the study titled Multi-tissue transcriptomic aging atlas reveals predictive aging biomarkers in the killifish, *ezh1* upregulated with age, while *ezh2* is downregulated across brain, fat, heart, and muscle.

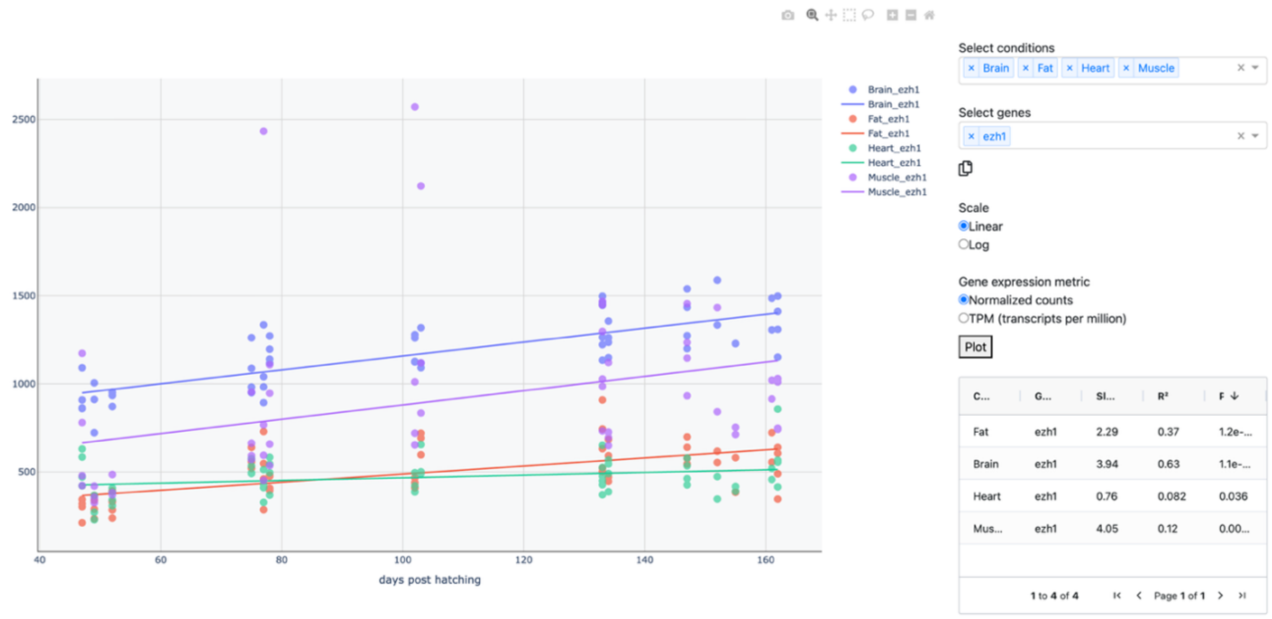

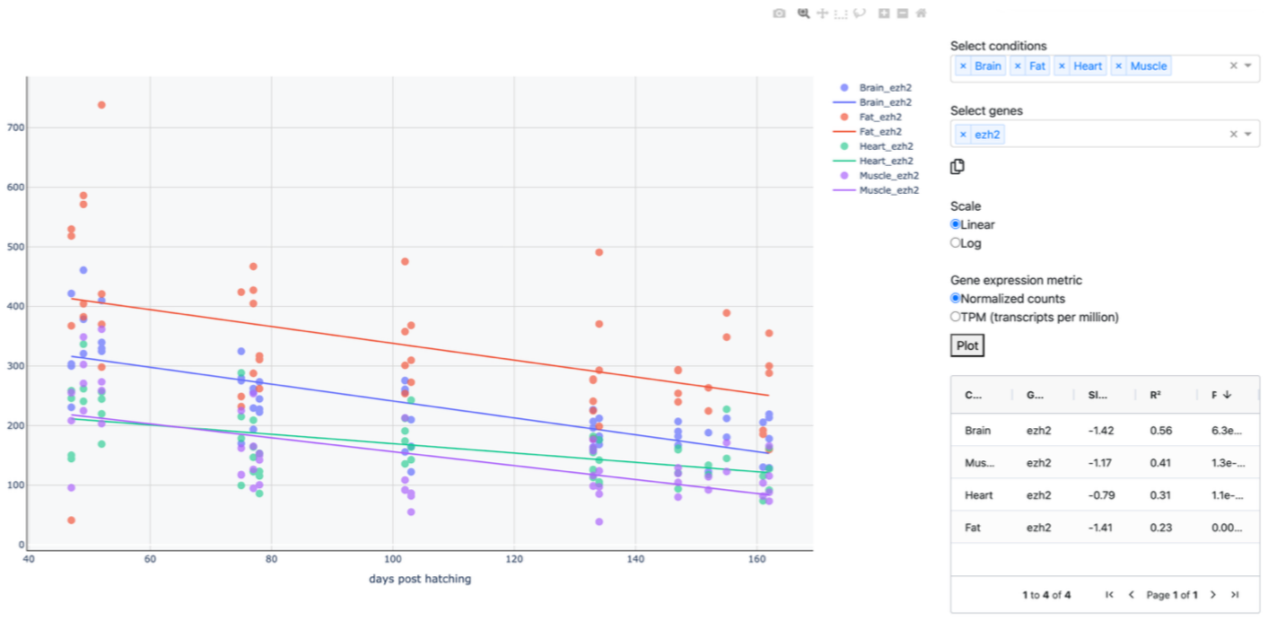

Figure S6. Line graph for the expression of *ezh1* and *ezh2* with age in male brain from *N. furzeri* (MZM-04/10) from study titled [RNA-seq of the aging brain in the short-lived fish N. furzeri - conserved pathways and novel genes associated with neurogenesis]

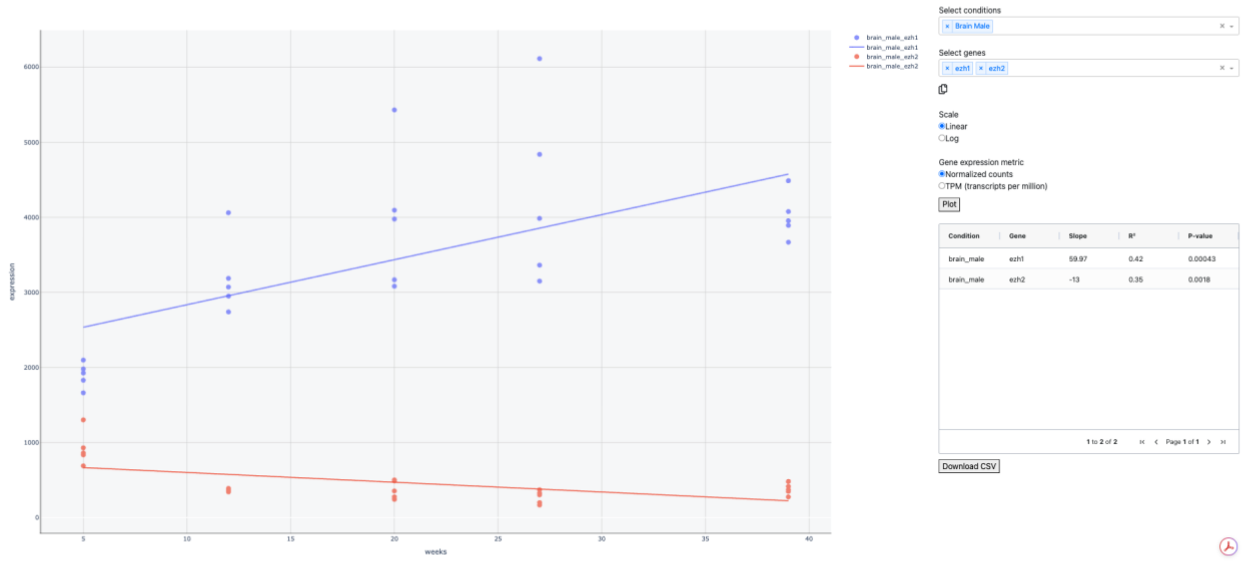

Figure S7. Information of *LOC107394210* gene in *N. furzeri* from Killiverse. This includes a brief description, GO annotations and links to NCBI Gene pages for orthologs from other model organisms.

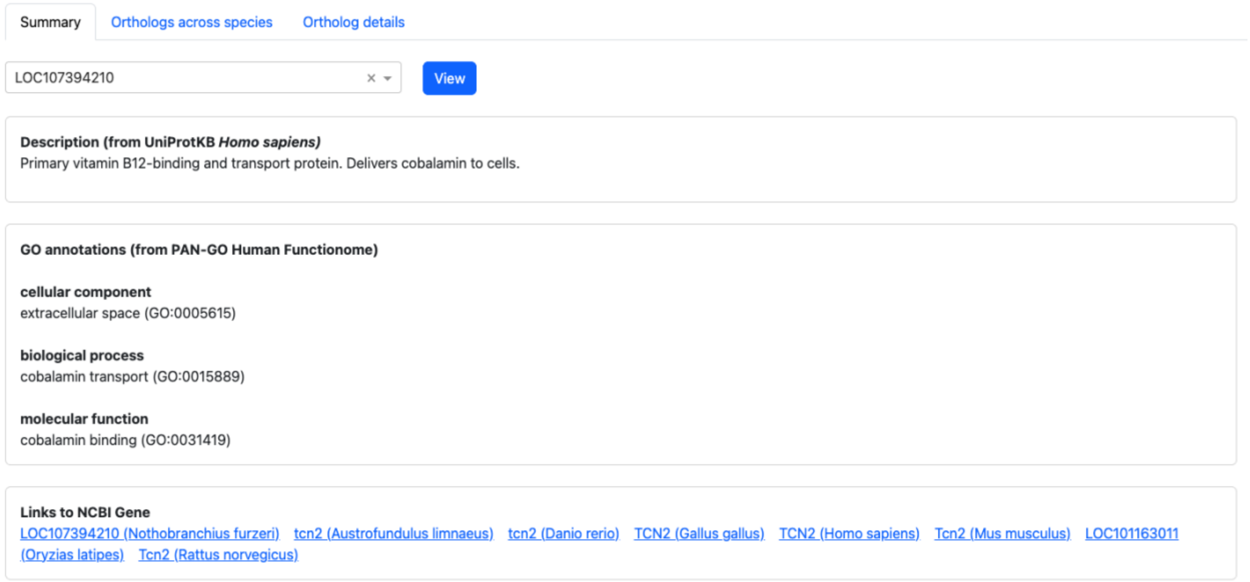

Figure S8. Line graph for the expression of *LOC107394210* with age in male brain from *N. furzeri* (GRZ) from study titled Analysis of different strains of the turquoise killifish Nothobranchius furzeri identifies transcriptomic signatures associated with heritable lifespan differences.

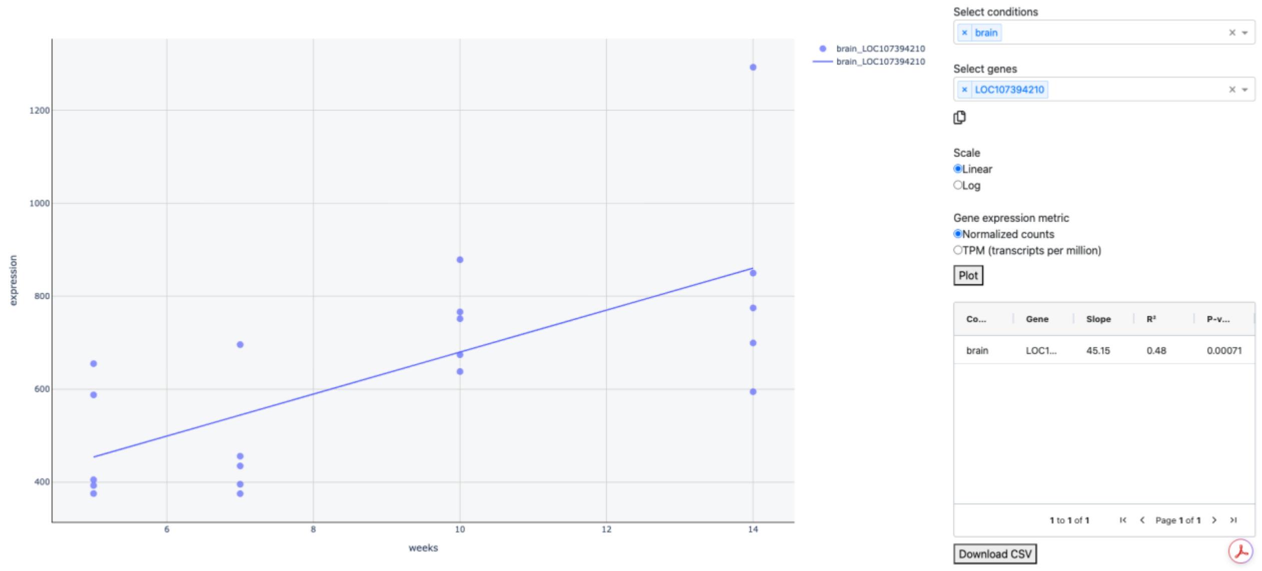

Figure S9. Spearman's rank correlation and adjusted p-value with age for *LOC107394210* expression. This data was obtained from *N. furzeri* (GRZ) in study titled Multi-tissue transcriptomic aging atlas reveals predictive aging biomarkers in the killifish.

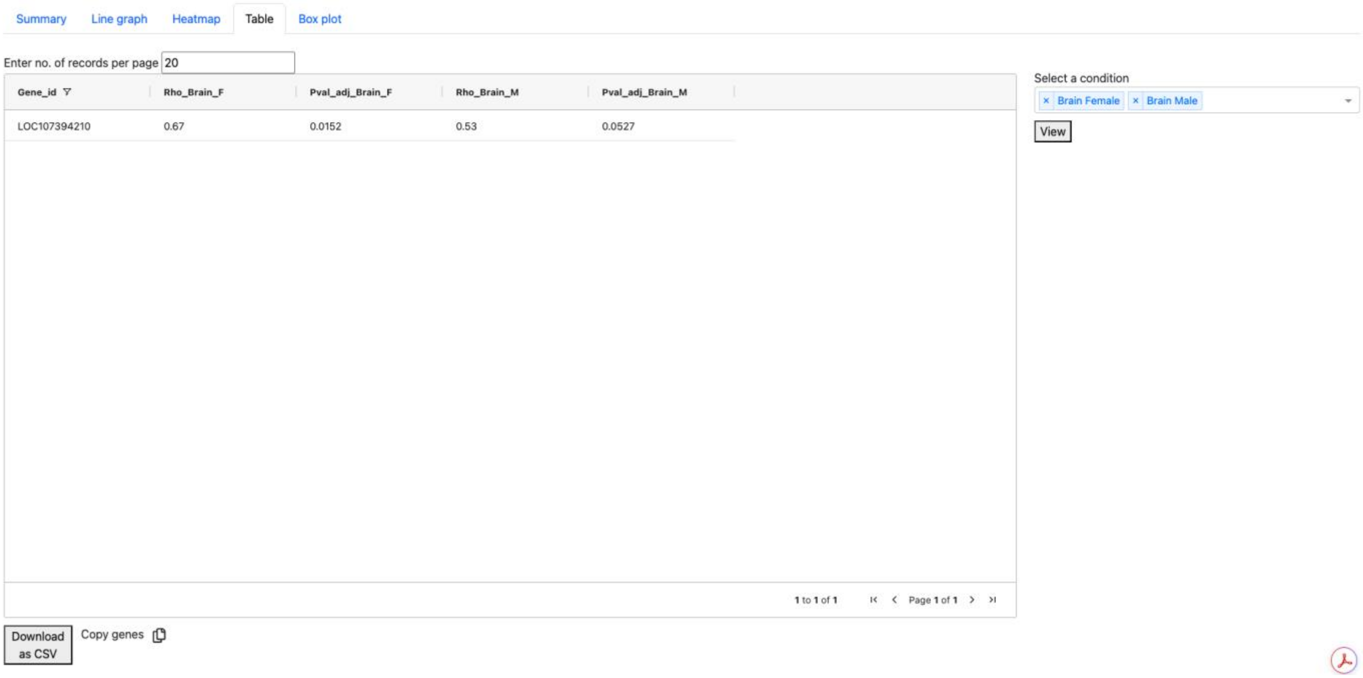

Gene_id	Rho_Brain_F	Pval_adj_Brain_F	Rho_Brain_M	Pval_adj_Brain_M
LOC107394210	0.67	0.0152	0.53	0.0527

Figure S10. Line graph for the expression of *LOC107394210* with age in male brain from *N. furzeri* (MZM-04/10) from study titled RNA-seq of the aging brain in the short-lived fish N. furzeri - conserved pathways and novel genes associated with neurogenesis

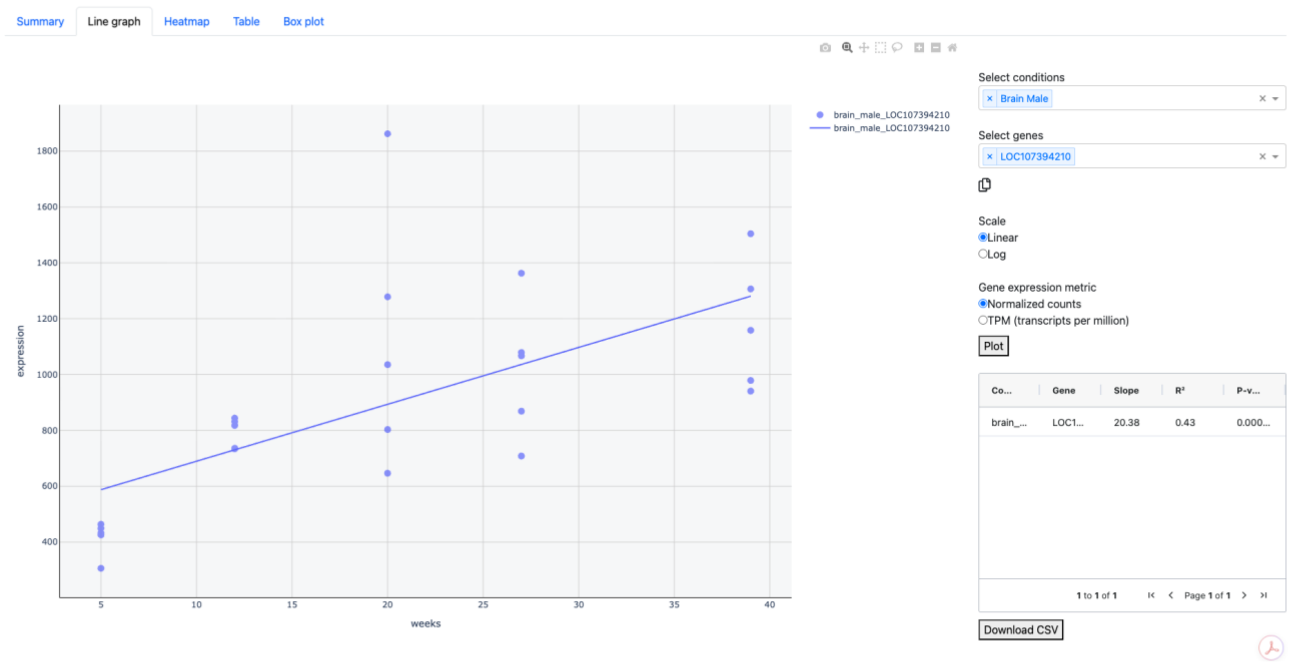

Figure S11. Box plot for the expression of *LOC107394210* with age in male and female brain from *N. guentheri* (Zanzibar TAN 14-02) from study titled The effect of meclofenoxate on the transcriptome of aging brain of Nothobranchius guentheri annual killifish

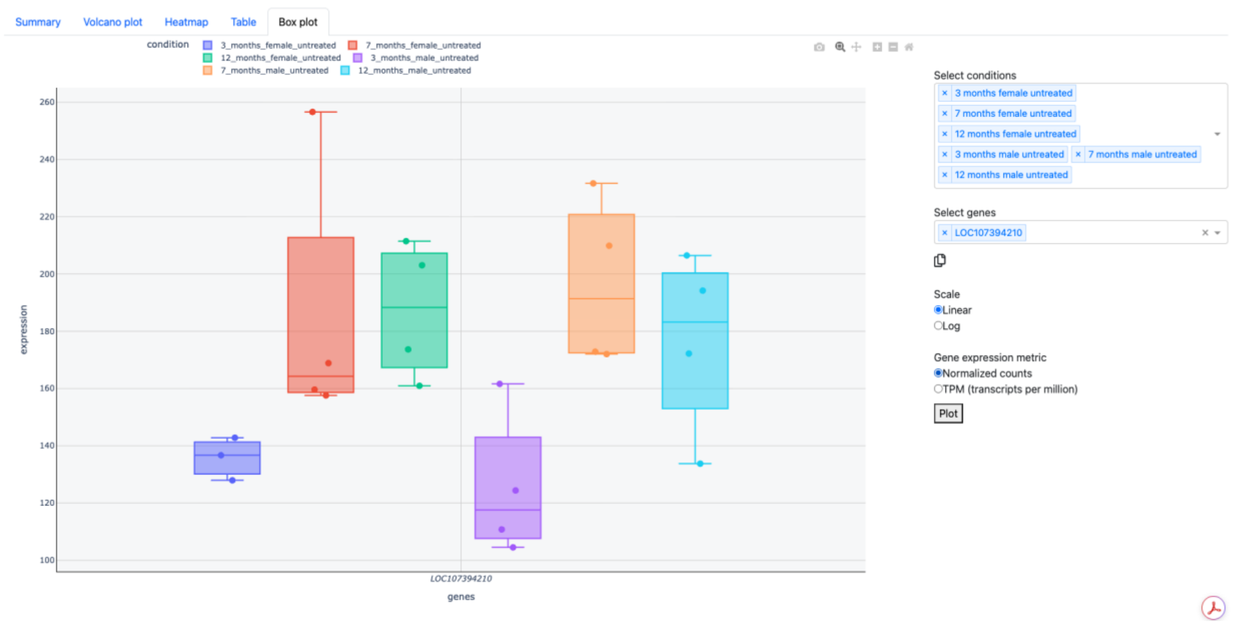

Figure S12. Expression of LOC107394210 in total lysate proteome data from young (3.5 months) and old (7 months) male brain of *N. furzeri* (GRZ), from the study Tissue-specific landscape of protein aggregation and quality control in an aging vertebrate (Dataset 2). Difference is not statistically significant (p-value = 0.25)

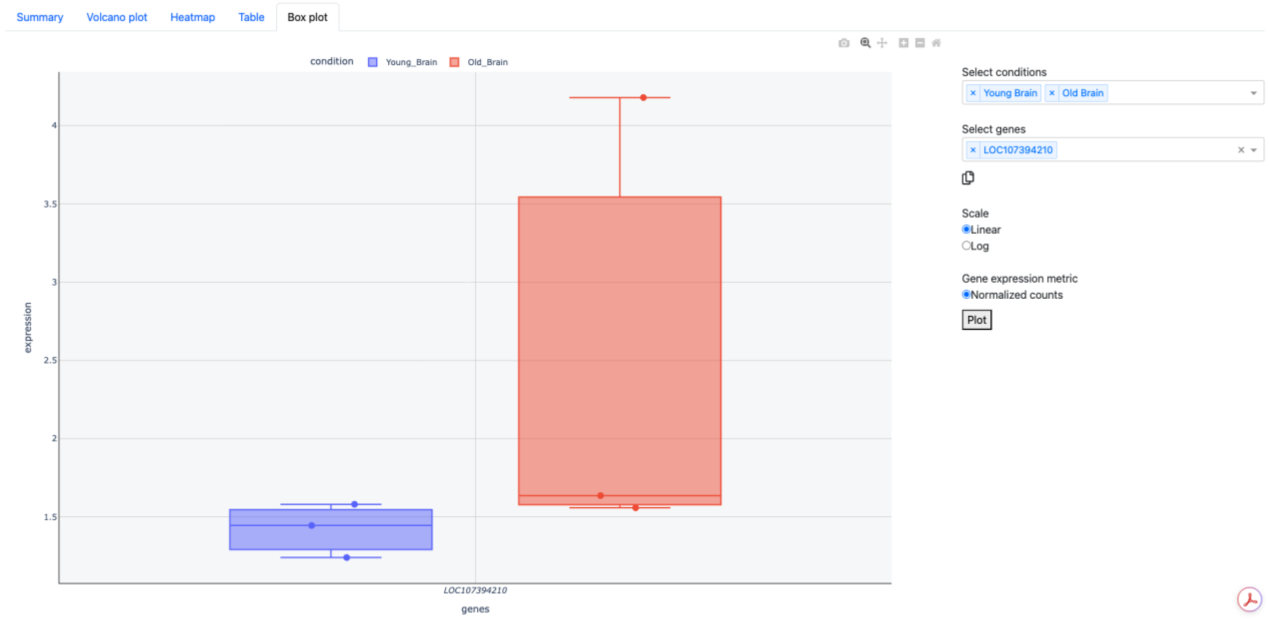

Figure S13. UMAP plot for single nucleus RNA-seq dataset from brain in study titled A multi-omic atlas in the African turquoise killifish reveals increased glucocorticoid signaling as a hallmark of brain aging. The points are colored by cell type.

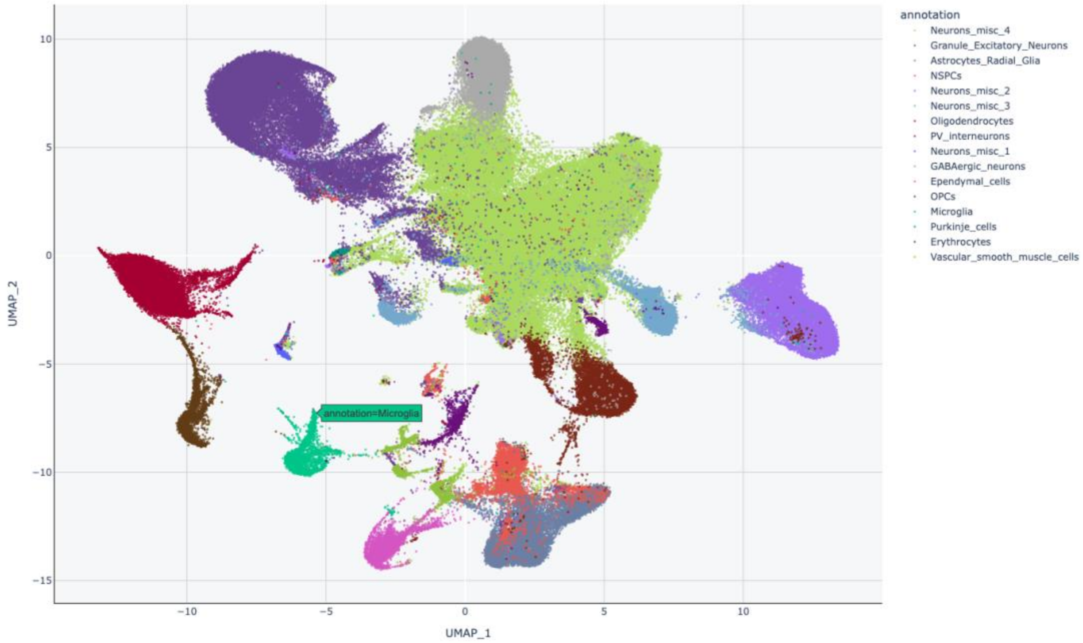

Figure S14. UMAP plot for single nucleus RNA-seq dataset from brain in study titled A multi-omic atlas in the African turquoise killifish reveals increased glucocorticoid signaling as a hallmark of brain aging. The points are colored by expression for *LOC107394210*.

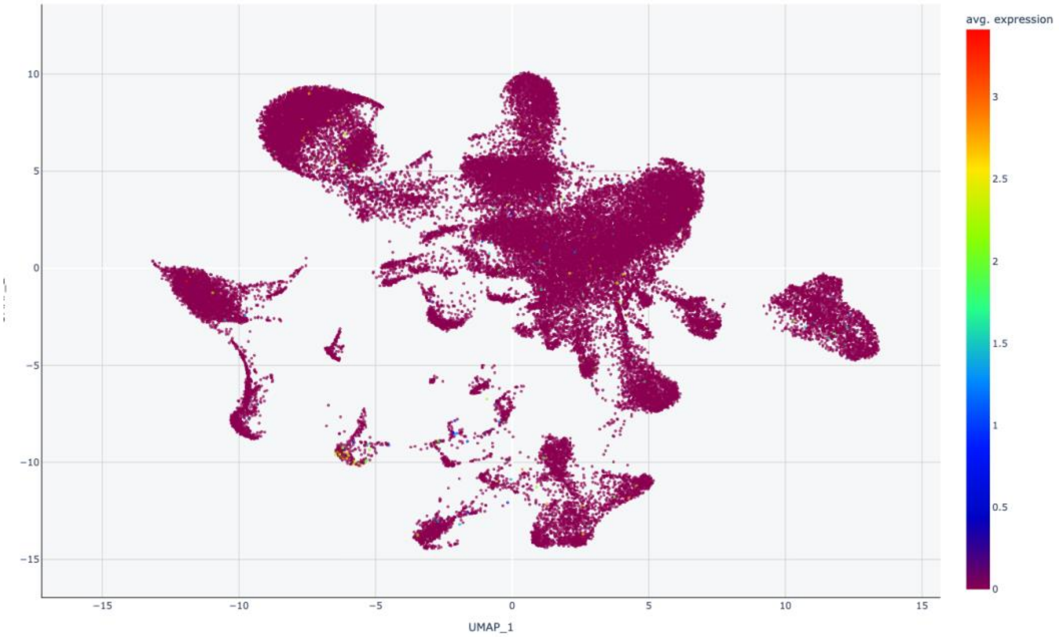

Figure S15. Bubble plot for single nucleus RNA-seq dataset from brain in study titled A multi-omic atlas in the African turquoise killifish reveals increased glucocorticoid signaling as a hallmark of brain aging. Bubble size represents the fraction of cells with non-zero expression of *LOC107394210*, while color indicates the average expression level among cells with non-zero expression.

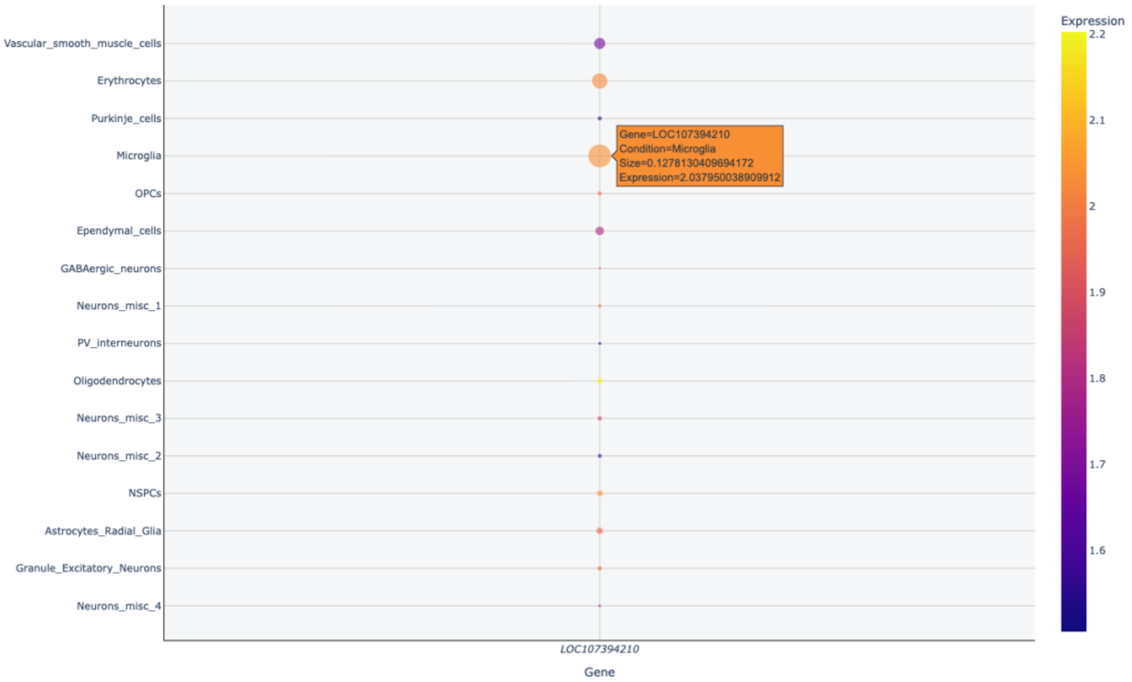

Supplement: Supplement 1 [file media-1.pdf]
